# Relationship Between Triglyceride–Glucose Index and Handgrip Strength in the Midlife and Elderly Population: Evidence From a Chinese Cohort

**DOI:** 10.1155/ije/8838404

**Published:** 2025-10-24

**Authors:** Zhaoliang Zhang, Yunfei Xu, Liehui Yao, Tao Ma, Liming Zhou

**Affiliations:** Orthopedic Department, The Affiliated YiXing Hospital of Jiangsu University, Ward 108, Yixing 214200, Jiangsu, China

**Keywords:** CHARLS, handgrip strength, insulin resistance, sarcopenia, triglyceride–glucose index

## Abstract

**Aims:**

At present, only a limited number of studies have investigated the association between muscle strength and insulin resistance. This study aimed to investigate the relationship between handgrip strength, a recognized indicator of overall muscle function and healthy aging, and triglyceride–glucose (TyG)–based indicators, including the TyG index, TyG–body mass index (TyG-BMI), TyG–waist circumference (TyG-WC), and TyG–waist-to-height ratio (TyG-WHtR), among middle-aged and older Chinese adults.

**Methods:**

We utilized a cohort from the China Health and Retirement Longitudinal Study (CHARLS) collected in 2011 and 2015, comprising a total of 3318 participants. Handgrip strength was assessed using absolute handgrip strength (measured by dynamometers), relative handgrip strength (absolute handgrip strength standardized by BMI), and defined weak handgrip strength. The relationships between the TyG index and its derived measures (TyG-BMI, TyG-WC, and TyG-WHtR) and different grip strength outcomes were examined using both multivariable logistic and linear regression approaches. To evaluate potential nonlinear patterns, restricted cubic spline (RCS) models were applied. Supplementary and robustness analyses encompassed receiver operating characteristic (ROC) curve evaluation, stratification by quartiles, subgroup comparisons, and handling of missing data via multiple imputation.

**Results:**

Between 2011 and 2015, 610 participants developed weak handgrip strength. Among all TyG indices, only TyG-WHtR was significantly associated with grip strength indicators (absolute: *β* = −0.95, 95% CI: −1.56 and −0.33; relative: *β* = −0.06, 95% CI: −0.09 and −0.03; weak: OR = 1.34, 95% CI: 1.03 and 1.74). These associations remained robust when using cumulative TyG-WHtR. K-means clustering identified three TyG-WHtR trajectory subgroups. Compared to the stable low group, both moderate (*β* = −0.10) and sharply increasing groups (absolute: *β* = −1.19; relative: *β* = −0.15) showed a greater risk of muscle decline. ROC curves indicated similar diagnostic accuracy for baseline and cumulative TyG-WHtR.

**Conclusions:**

Higher TyG-WHtR levels appear to be independently linked to poorer handgrip strength performance in midlife and elder adults. Maintaining a low TyG-WHtR may contribute to improving the health status of midlife and elderly adults by preserving handgrip strength.

## 1. Background

The aging process is often accompanied by progressive changes in muscle mass and strength. Research indicates that after the age of 50, the loss of muscle strength per decade is approximately 15% greater than before the age of 50 [1]. Handgrip strength, as the most direct and convenient measure of muscle strength, is considered one of the key biomarkers of aging and frailty [2]. Reduced handgrip strength has been associated with a wide range of adverse health outcomes, including falls, bone fractures, nutritional deficiencies, obesity, cardiovascular diseases, and metabolic syndromes [3–5]. Thus, handgrip strength is recognized as a strong predictor of disability, morbidity, and mortality in middle-aged and older adults [6]. Over the past 2 decades, global data show a declining trend in handgrip strength across various populations [7]. The analysis of weak handgrip strength has important implications for public health, as it may help extend life expectancy and support preventive strategies for middle-aged and older adults worldwide.

Insulin resistance (IR) is considered one of the major mechanisms underlying decreased muscle strength [8]. Studies show a higher prevalence of sarcopenia among patients with Type 2 diabetes [9]. Hyperglycemia-associated oxidative stress and chronic inflammation are key factors leading to an imbalance between muscle protein synthesis and breakdown. In addition, IR may interfere with the normal energy metabolism of muscle cells [10, 11]. The triglyceride–glucose (TyG) index (TyG index), calculated from fasting triglyceride and fasting glucose, is widely recognized as a practical surrogate marker for IR [12]. Moreover, the TyG index is frequently combined with obesity-related indicators such as body mass index (BMI), waist circumference (WC), and waist-to-height ratio (WHtR), resulting in TyG-BMI, TyG-WC, and TyG-WHtR, which may provide greater specificity in disease risk assessment [13, 14]. Chen et al. [15] observed an inverse relationship between the TyG index and the prevalence of sarcopenia among older Chinese adults; however, this association lost statistical significance after adjustment for BMI. To date, few studies have examined the associations between these TyG-derived indices and muscle strength, and the evidence remains limited.

The aim of this study is to investigate the associations of the TyG index, TyG-BMI, TyG-WC, and TyG-WHtR with handgrip strength using data from the China Health and Retirement Longitudinal Study (CHARLS), a nationally representative cohort. We hypothesize that certain TyG-based indices may better predict the decline in handgrip strength among middle-aged and older adults.

## 2. Methods

### 2.1. Study Population

The participants in this study were collected from CHARLS, which includes data on physical health, socioeconomic status, psychological assessments, and physical functioning tests of middle-aged and older adults in China, obtained through proportional multistage sampling. CHARLS conducts regular follow-up surveys on participants every 2 or 3 years [16]. CHARLS complies with the Declaration of Helsinki and was approved by the Ethics Committee of Peking University. All participants had provided informed consent and signed the relevant consent forms [16].

**Figure 1:**
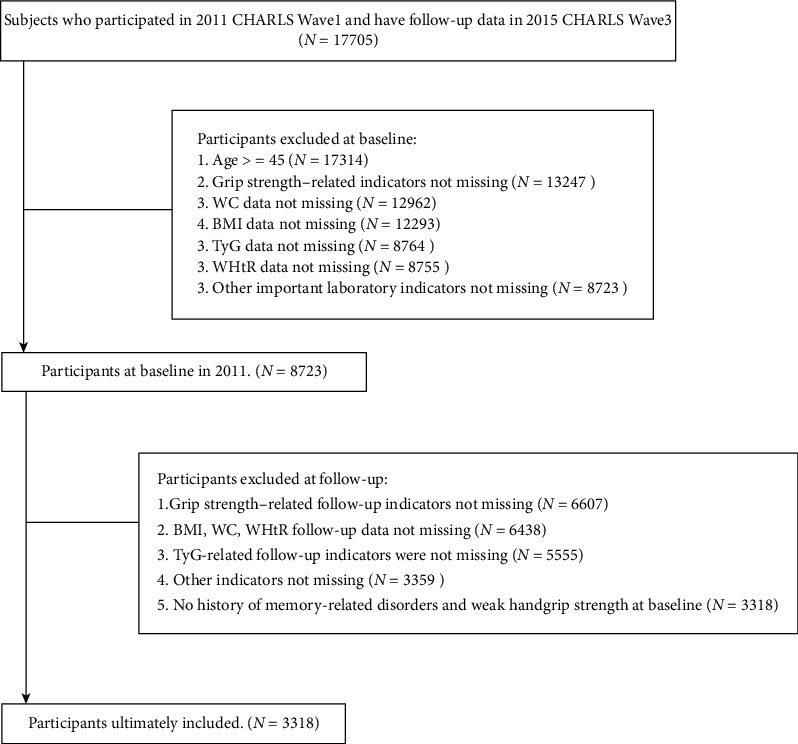
Flowchart of the participants' selection process.

Our study used 2011 as the baseline and handgrip strength at the end of follow-up in 2015 as the outcomes. We excluded individuals who met any of the following conditions: (1) age below 45 years, (2) missing information on handgrip or inability to complete relevant tests, (3) lack of other necessary variables, and (4) presence of mental or memory-related diseases or weak handgrip strength at baseline. Ultimately, this analysis included 3318 participants (Figure 1).

### 2.2. Handgrip Strength Assessment

The absolute handgrip strength of participants was measured as absolute handgrip strength using the TMWL-1000 dynamometer (manufactured by Nantong Yuejian Physical Measurement Instrument Co., Ltd). Participants' dominant handgrip strength was measured twice, and the mean value of the two grip strength readings was used to represent absolute handgrip strength. Relative handgrip strength was calculated by using the following formula: absolute handgrip strength (kg)/BMI (kg/m^2^) [5, 17]. Weak muscle strength was defined according to sex-specific thresholds: < 18 kg for women and < 28 kg for men [18, 19].

### 2.3. Calculation and Integration of TyG-Derived Indicators

The standard TyG index was computed by using the following formula: ln (fasting triglycerides [mg/dL]) multiplied by fasting plasma glucose (mg/dL), then divided by 2 [14]. WHtR was calculated as follows: WC (cm) divided by height (cm). To construct composite metabolic markers, we used the following equations: TyG-WC = TyG × WC; TyG-WHtR = TyG × WHtR; and TyG-BMI = TyG × BMI [14, 20]. These indices were derived from laboratory test results at baseline and at the end of follow-up. In addition, to comprehensively assess the changes in participants' indices, we calculated the cumulative index as follows: cumulative index = (index in 2011 + index in 2015)/2  ×  (2015–2012) [13].

### 2.4. Covariates

Our primary covariates were drawn from 3 aspects: sociodemographic factors, health-related factors, and laboratory test results [18, 21]. Sociodemographic factors comprised age, gender, residence area (rural or urban), education level (secondary education or above vs. below), marital status (married/cohabitation vs. other), and insurance coverage (under insurance vs. no insurance). Health-related factors included BMI, smoking status, alcohol consumption, WC, WHtR, presence of hypertension, presence of diabetes, and presence of dyslipidemia. Laboratory test results included CRP, total cholesterol (TC), high-density lipoprotein cholesterol (HDL-C), HbA1c, BUN, and blood glucose. Individuals were classified as having hypertension if their systolic blood pressure was at least 140 mmHg, diastolic blood pressure was 90 mmHg or higher, or if they reported a physician-diagnosed history of hypertension. Diabetes was identified by a fasting plasma glucose level of ≥ 7.0 mmol/L or self-reported diagnosis of the condition. Dyslipidemia was identified based on any of the following criteria: TC ≥ 240 mg/dL, triglyceride levels ≥ 150 mg/dL, low-density lipoprotein cholesterol (LDL-C) ≥ 160 mg/dL, HDL-C < 40 mg/dL, or a self-reported history of lipid disorders [13, 22].

### 2.5. Statistical Analysis

Results for continuous variables are presented as mean ± standard deviation (M±SD) for normally distributed data and as median (interquartile range [IQR]) for non-normally distributed data. The normality of distributions was assessed using the Shapiro–Wilk test. Categorical variables were expressed as counts and percentages (*n*, %). Multivariable linear regression and multivariable logistic regression were used to examine the associations between different TyG indices and handgrip strength measures. Model 1 included age, marital status, insurance coverage, education, and residence. Model 2 included BMI, alcohol consumption, smoking status, hypertension, diabetes, and dyslipidemia. Model 3 included all covariates. Restricted cubic splines (RCSs) are used to describe dose–response relationships of continuous variables. K-means clustering analysis was used to perform appropriate clustering of the TyG indices from the two follow-up assessments. Multiple imputation (by the R package “mice”) was conducted for nonprimary variables with missing data below 30%, and then the analysis of the main results was repeated to verify the stability of the findings. Subgroup analyses were also conducted as part of the sensitivity test. In addition, for continuous variables, any values differing from the overall mean by more than three standard deviations (either above or below) were classified as outliers and treated as missing data. A *p* value of less than 0.05 was considered statistically significant. *p* values were denoted by the following symbols: “^∗^” for < 0.05, “^∗∗^” for < 0.01, and “^∗∗∗^” for < 0.001. All statistical procedures were executed using R (V4.4.1).

## 3. Results

### 3.1. Participant Characteristics

This study enrolled 3318 individuals, with a mean baseline age of 59.32 ± 8.95 years. Among them, 1656 (49.9%) were women. At the end of the follow-up period, 610 participants (18.4%) met the criteria for weak handgrip strength. Compared to participants with normal handgrip strength, those with weak handgrip strength generally had lower BMI and WC; higher levels of CRP, HDL-C, and BUN; and were more likely to have diabetes and hypertension. There were also statistically significant differences in the TyG indices between the two groups, except for TyG-WHtR. Specific basic characteristics are shown in Table 1.

### 3.2. TyG-WHtR Was Significantly Associated With Handgrip Strength

Table 2 displays the results of the multiple regression analyses. It could be found that **a**fter adjusting for all confounding variables, only TyG-WHtR showed a significant association with all 3 handgrip strength outcomes (absolute handgrip strength: beta = −0.95 [95% CI: −1.56 and −0.33], relative handgrip strength: beta = −0.06 [95% CI: −0.09 and −0.03], and weak handgrip strength: OR = 1.34 [95% CI: 1.03 and 1.74]) Therefore, we calculated only cumulative TyG-WHtR values in the subsequent analyses (Table 2). Regression analyses revealed cumulative TyG-WHtR as an independent risk factor for reduced handgrip strength as well (absolute handgrip strength: beta = −0.28 [95% CI: −0.44 and −0.12], relative handgrip strength: beta = −0.03 [95% CI: −0.04 and −0.03], and weak handgrip strength: OR = 1.11 [95% CI: 1.03 and 1.18]).

**Figure 2:**
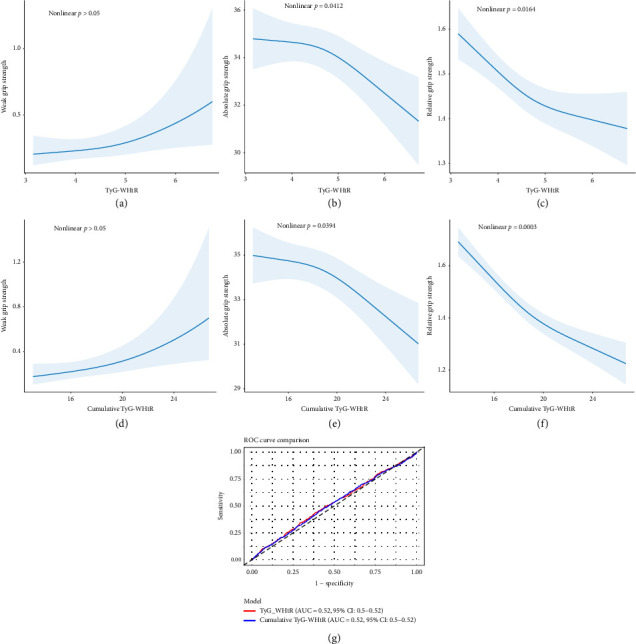
Association between TyG-WHtR, cumulative TyG-WHtR, and outcome measures analyzed using RCS. (a–f) ROC curves showed that TyG-WHtR and cumulative TyG-WHtR had similar diagnostic capabilities for identifying low handgrip strength. (g) Model 3 was used for adjusting confounding factors.

The RCS regression model showed a nonlinear decrease in TyG-WHtR in relation to absolute grip strength (nonlinear *p*=0.0412) and relative grip strength (nonlinear *p*=0.0164) (Figures 2(b) and 2(c)). Besides, a similar trend was found in cumulative TyG-WHtR (Figures 2(e) and 2(f)). However, their effects on weak handgrip strength are considered to be linear (Figures 2(a) and 2(d)). To compare the diagnostic abilities of these 2 indices for weak handgrip strength, we plotted the receiver operating characteristic (ROC) curves based on logistic regression results (Figure 2(g)). The curves indicated that both indices had similar diagnostic performance (AUC = 0.52, 95% CI: 0.50–0.54).

We then converted TyG-WHtR and cumulative TyG-WHtR into quartiles of continuous variables (IQR) and performed supplementary analyses (Table 3). We used the first quartile as the reference. We found that participants in the fourth quartile (Q4) of TyG-WHtR had lower absolute handgrip strength (beta = −1.61 [95% CI: −2.73 and −0.50], *p* for trend < 0.001), and similar results were observed for cumulative TyG-WHtR (beta = −1.51[95% CI: −2.62 and −0.40], *p* for trend < 0.05). However, participants in the Q2 (beta = −0.06 [95% CI: −0.09 and −0.03]), Q3 (beta = −0.09 [95% CI: −0.13 and −0.05]), and Q4 (beta = −0.11[95% CI: −0.16 and −0.06]) of TyG-WHtR all exhibited lower relative handgrip strength (*p* for trend < 0.001). Similarly, participants in the Q2 (beta = −0.11 [95% CI: −0.14 and −0.08]), Q3 (beta = −0.15 [95% CI: −0.19 and −0.11]), and Q4 (beta = −0.21[95% CI: −0.25 and −0.16]) of cumulative TyG-WHtR also had lower levels of relative handgrip strength (*p* for trend < 0.001). Interestingly, only participants in the Q4 of cumulative TyG-WHtR were more likely to be identified as suffering from weak handgrip strength (beta = −1.63 [95% CI: 1.01, 2.62], *p* for trend < 0.05).

### 3.3. Higher and Rapidly Increasing TyG-WHtR Was Associated With Lower Handgrip Strength

**Figure 3:**
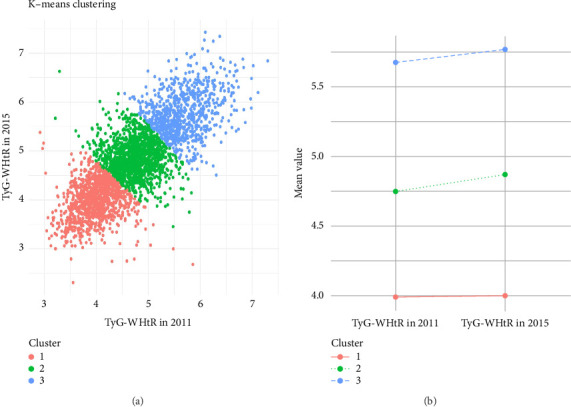
Clustering analysis results of participants based on K-means using TyG-WHtR measurements from 2011 and 2015.

To comprehensively assess whether changes in TyG-WHtR over the 4-year period had an impact on handgrip strength, we performed K-means clustering on participants based on their TyG-WHtR in 2011 and 2015. As a result, we identified 3 distinct clusters (Figure 3(a)). We found that Cluster 1 was characterized by consistently low and stable TyG-WHtR over the 2 years, Cluster 2 showed moderate levels, while Cluster 3 had high TyG-WHtR with a marked increasing trend (Figure 3(b)).

We then conducted regression analysis using Cluster 1 as the reference, and the results were consistent with our hypothesis (Table 4). Compared to Cluster 1, participants in Cluster 3 had not only lower absolute handgrip strength (beta = −1.19 [95% CI: −2.21 and −0.16]) but also lower relative handgrip strength (beta = −0.15 [95% CI: −0.20 and −0.11]). Participants in Cluster 2 were only found to have lower relative handgrip strength (beta = −0.10 [95% CI: −0.13 and −0.07]). Neither Cluster 2 nor Cluster 3 showed a significantly increased risk of weak handgrip strength compared to Cluster 1.

### 3.4. Stratified and Sensitivity Analyses

Table 5 showed the correlation between TyG-WHtR and cumulative TyG-WHtR with handgrip strength, stratified by different factors. Notably, TyG-WHtR did not appear to be associated with handgrip strength among diabetic patients. The remaining subgroups showed a degree of consistency with the analysis of the overall population. In addition, analysis of the data after multiple imputation also yielded results consistent with the direction of the findings (See Supplementary Table in Supporting Information [available here]). This confirmed the reliability of our study.

## 4. Discussion

This analysis explored the relationships of four distinct TyG indices with handgrip strength among midlife and elderly individuals. Upon full covariate adjustment, TyG-WHtR emerged as the only index potentially linked independently to reduced muscular strength. Both baseline and cumulative 4-year TyG-WHtR showed similar diagnostic performance for weak handgrip strength. RCS and quartile analyses indicated that only high levels of TyG-WHtR were associated with lower absolute and relative handgrip strength, while no significant association was observed for the defined weak handgrip strength. Furthermore, maintaining a low and stable TyG-WHtR over the 4-year follow-up period was associated with the preservation of handgrip strength.

Few studies to date have examined how the TyG index correlates with muscle strength, but IR is widely recognized as being closely related to muscle strength [23–26]. Moon SS et al. [27] reported a notable correlation between sarcopenia and HOMA-IR in a nonobese population within a Korean cohort. Furthermore, sarcopenia was considered a contributing factor in the onset of diabetes within this population. Recognized as a robust indicator of IR, the TyG index was shown in a cohort study by Son DH et al. [28] to have superior predictive power for metabolic syndrome when compared with HOMA-IR (AUC: 0.837 vs. 0.680, *p* < 0.001). Besides, the calculation of TyG requires only glucose and triglyceride levels, whereas other IR measures such as HOMA-IR may not be readily available for population screening. Therefore, the TyG index also has an irreplaceable advantage in terms of convenience [29]. Other derived indices of TyG were considered to be more successful in identifying IR than TyG alone [30]. Ren Q et al. [13] found that not only baseline TyG-WHtR but also an increase in TyG-WHtR were risk factors for new-onset cardiovascular disease in midlife and elder adults. Zhang X et al. [14] compared the associations between different TyG indices and arthritis, finding that TyG-BMI and TyG-WHtR were positively correlated with the prevalence of arthritis in both Chinese and American populations, showing higher diagnostic capability compared to TyG. To our knowledge, this study is the first to examine the relationship between various TyG-related indices and muscle strength, providing new evidence for the link between IR and sarcopenia. Further randomized controlled trials are warranted to clarify underlying mechanisms and establish causality.

Given that TyG-WHtR is a time-varying metric, evaluating both baseline and follow-up measures is important to capture its clinical relevance. In our study, we introduced the cumulative TyG-WHtR index, which has not yet been explored in studies on the same topic [13, 31]. Participants were categorized into three distinct subgroups using the K-means clustering algorithm, according to their TyG-WHtR trajectories between 2011 and 2015. An additional commentary arises from our clustering analysis. By identifying three distinct TyG-WHtR trajectories, we show that not only absolute levels but also longitudinal changes in TyG-WHtR carry clinical relevance. Participants with high and rapidly increasing TyG-WHtR were at the greatest risk of muscular decline. This dynamic perspective suggests that monitoring TyG-WHtR over time may be more informative than relying on single baseline measurements. Such findings support the need for routine, repeated assessment of simple biomarkers such as TyG-WHtR in clinical and community settings to identify individuals at risk of functional decline before overt sarcopenia develops.

Previous research had emphasized that an excessive WC, indicative of obesity, could lead to decreased handgrip strength in older adults [32, 33]. This is consistent with our study's findings. The relationship between the TyG index and diminished muscle strength may be interpreted from two perspectives when considered alongside obesity indicators. On one hand, an increase in fat mass directly leads to elevated levels of associated metabolites and proinflammatory factors, which can directly impair skeletal muscle cells [34, 35]. On the other hand, IR mediates a reduction in protein synthesis metabolism, including impairing muscle protein synthesis [36]. Furthermore, in the subgroup analysis, the association between TyG-WHtR and handgrip strength was more pronounced in the nondiabetic group compared to the diabetic group. We hypothesize that this phenomenon may be attributable to the more severe IR in diabetic patients. Additional studies are warranted to deepen our understanding in this field.

From a public health perspective, our findings highlight the potential utility of TyG-WHtR in screening and prevention strategies. Since its calculation requires only fasting glucose and triglyceride levels, commonly available in routine health checks, it represents a cost-effective and scalable tool for risk stratification. Incorporating TyG-WHtR into preventive care models may help identify vulnerable individuals, enabling early lifestyle or pharmacological interventions aimed at preserving muscle strength and delaying frailty.

Our study has the following limitations: First, since our study sample comprises Asian individuals, the applicability of these results to other ethnic populations should be interpreted with caution. Second, we excluded participants who were unable to complete the handgrip strength test, which may introduce a certain degree of bias. There may have been loss to follow-up due to participant mortality, which could potentially influence the conclusions due to the impact of competing events [13]. Finally, the cohort study with follow-up provides insights into the correlation between TyG-WHtR and handgrip strength from multiple perspectives, but we are unable to elucidate the underlying biological mechanisms. Besides, we did not exclude individuals with neurological disorders, rheumatologic conditions, malignancies, myopathies, or herniated discs that may affect grip strength. This decision was partly due to the limited sample size and the absence of standardized exclusion criteria in the dataset. In addition, previous studies in related fields have not consistently excluded such populations; we were concerned that arbitrary exclusions might introduce new sources of bias rather than reduce them [37–39]. However, our study still offers valuable insights.

## 5. Conclusions

In our study, we found that higher and progressively increasing TyG-WHtR values were associated with decreased handgrip strength in older adults. In individuals who have not yet experienced a decrease in handgrip strength but have high TyG-WHtR values, this finding may guide timely intervention to reduce the risk of future handgrip strength decline. Prospective studies involving larger populations and fewer confounding factors are needed to clarify this finding.

## Figures and Tables

**Table 1:** Characteristics of the participants.

	Overall	Normal grip strength	Weak grip strength	*p* value
*n*	3318	2708	610	
Gender = female (*n*, %)	1656 (49.9)	1395 (51.5)	261 (42.8)	< 0.001
Age (years, mean [SD])	59.32 (8.95)	57.98 (8.30)	65.24 (9.33)	< 0.001
Education = secondary education or above (*n*, %)	46 (1.4)	43 (1.6)	3 (0.5)	0.057
Residence = urban (*n*, %)	521 (15.7)	473 (17.5)	48 (7.9)	< 0.001
Marital status = married/cohabitation (*n*, %)	2735 (82.4)	2280 (84.2)	455 (74.6)	< 0.001
Under insurance (*n*, %)	3148 (94.9)	2568 (94.8)	580 (95.1)	0.878
Drinking = True (*n*, %)	1104 (33.3)	917 (33.9)	187 (30.7)	0.141
Smoking = True (*n*, %)	1377 (41.5)	1097 (40.5)	280 (45.9)	0.017
BMI (Kg/m^2^, mean [SD])	23.62 (3.38)	23.82 (3.35)	22.77 (3.37)	< 0.001
Hypertension (*n*, %)	1369 (41.5)	1076 (40.0)	293 (48.3)	< 0.001
Diabetes (*n*, %)	1469 (44.3)	1223 (45.2)	246 (40.3)	0.033
Dyslipidemia (*n*, %)	178 (5.4)	148 (5.5)	30 (5.0)	0.665
WC (cm, mean [SD])	85.73 (9.56)	86.03 (9.50)	84.37 (9.69)	< 0.001
WHtR (mean [SD])	0.54 (0.06)	0.54 (0.06)	0.54 (0.07)	0.607
CRP (mg/L, mean [SD])	1.88 (2.76)	1.81 (2.64)	2.23 (3.23)	0.001
Glucose (mg/dL, mean [SD])	106.12 (21.07)	105.86 (20.99)	107.29 (21.43)	0.135
TC (mmol/L, mean [SD])	4.99 (0.92)	4.99 (0.91)	4.98 (0.93)	0.868
HDL-C (mmol/L, mean [SD])	1.31 (0.36)	1.30 (0.36)	1.34 (0.38)	0.019
HbA1c (%, mean [SD])	5.19 (0.50)	5.19 (0.51)	5.17 (0.50)	0.397
BUN (mg/dL, mean [SD])	15.55 (4.10)	15.45 (4.04)	16.01 (4.33)	0.002
TyG (mean [SD])	8.68 (0.61)	8.69 (0.62)	8.63 (0.58)	0.032
TyG-WC (mean [SD])	745.73 (112.37)	749.21 (111.82)	730.29 (113.59)	< 0.001
TyG-BMI (mean [SD])	205.59 (36.60)	207.48 (36.35)	197.21 (36.57)	< 0.001
TyG-WHtR (mean [SD])	4.71 (0.73)	4.72 (0.73)	4.68 (0.77)	0.218
Cumulative TyG-WHtR (mean [SD])	19.01 (2.84)	19.05 (2.81)	18.84 (2.96)	0.099
Absolute grip strength (kg, mean [SD])	29.64 (9.30)	31.97 (8.27)	19.31 (6.01)	< 0.001
Relative grip strength (kgf/kg, mean [SD])	1.27 (0.43)	1.36 (0.40)	0.87 (0.32)	< 0.001

**Table 2:** Results of the multivariable logistic regression and multivariable linear regression for different TyG indices and handgrip strength measures.

	**Absolute grip strength (*β*-coefficients [95% CI])**		
	**Model 1**	**Model 2**	**Model 3**

TyG	0.77 (0.38, 1.15)^∗∗∗^	0.16 (−0.33, 0.64)	−0.09 (−0.64, 0.46)
TyG-BMI	0.03 (0.02, 0.03)^∗∗∗^	0.00 (−0.02, 0.02)	−0.01 (−0.03, 0.02)
TyG-WC	0.01 (0.01, 0.01)^∗∗∗^	0.01 (0.00, 0.01)^∗∗∗^	0.02 (0.00, 0.01)^∗∗∗^
TyG-WHtR	0.84 (0.50, 1.18)^∗∗∗^	−0.73 (−1.30, −0.17)^∗^	−0.95 (−1.56, −0.33)^∗∗^
Cumulative TyG-WHtR	0.21 (0.12, 0.30)^∗∗∗^	−0.23 (−0.38, −0.08)^∗∗^	−0.28 (−0.44, −0.12)^∗∗∗^

	**Relative grip strength** (***β*-coefficients** [**95% CI])**		
	**Model 1**	**Model 2**	**Model 3**

TyG	−0.04 (−0.06, −0.03)^∗∗∗^	−0.00 (−0.02, 0.02)	−0.01 (−0.03, 0.02)
TyG-BMI	−0.00 (−0.00, −0.00)^∗∗∗^	−0.00 (−0.00, 0.00)	−0.00 (−0.00, 0.00)
TyG-WC	0.00 (−0.00, −0.00)	−0.00 (−0.00, 0.00)	−0.00 (−0.00, 0.00)
TyG-WHtR	−0.13 (−0.15, −0.12)^∗∗∗^	−0.05 (−0.08, −0.03)^∗∗∗^	−0.06 (−0.09, −0.03)^∗∗∗^
Cumulative TyG-WHtR	−0.04 (−0.05, −0.04)^∗∗∗^	−0.03 (−0.04, −0.03)^∗∗∗^	−0.03 (−0.04, −0.03)^∗∗∗^

	**Weak grip strength** (**OR** [**95% CI])**		
	**Model 1**	**Model 2**	**Model 3**

TyG	0.86 (0.730, 1.00)	0.94 (0.77, 1.16)	0.98 (0.77, 1.24)
TyG-BMI	0.99 (0.99, 0.99)^∗∗^	0.99 (0.99, 1.01)	1.00 (0.98, 1.01)
TyG-WC	0.99 (0.98, 1.00)^∗∗^	0.99 (0.99, 1.00)	0.99 (0.99, 1.00)
TyG-WHtR	0.93 (0.81, 1.07)	1.29 (1.03,1.64)^∗^	1.34 (1.03, 1.74)^∗^
Cumulative TyG-WHtR	0.99 (0.95, 1.02)	1.09 (1.02, 1.16)^∗∗^	1.11 (1.03, 1.18)^∗∗^

*Note:p* values are denoted by the following symbols: “^∗^” for < 0.05, “^∗∗^” for < 0.01, and “^∗∗∗^” for < 0.001.

**Table 3:** Regression analysis results of TyG-WHtR and cumulative TyG-WHtR by IQR with handgrip strength outcomes.

**TyG-WHtR per IQR**	**Absolute grip strength (*β*-coefficients [95% CI])**		
	**Model 1**	**Model 2**	**Model 3**

Q1	Ref	Ref	Ref
Q2	0.68 (0.02, 1.34)^∗^	−0.16 (−0.85, 0.53)	−0.21 (−0.93, 0.51)
Q3	1.12 (0.45, 1.79)^∗∗^	−0.56 (−1.36, 0.25)	−0.74 (−1.59, 0.12)
Q4	1.58 (0.89, 2.27)^∗∗∗^	−1.31 (−2.35, −0.26)^∗^	−1.61 (−2.73, −0.50)^∗∗^
P for trend	< 0.001	< 0.05	< 0.001

**TyG-WHtR per IQR**	**Relative grip strength** (***β*-coefficients** [**95% CI])**		
	**Model 1**	**Model 2**	**Model 3**

Q1	Ref	Ref	Ref
Q2	−0.11 (−0.14, −0.08)^∗∗∗^	−0.06 (−0.09, −0.03)^∗∗∗^	−0.06 (−0.09, −0.03)^∗∗∗^
Q3	−0.18 (−0.21, −0.15)^∗∗∗^	−0.08 (−0.12, −0.05)^∗∗∗^	−0.09 (−0.13, −0.05)^∗∗∗^
Q4	−0.26 (−0.29, −0.23)^∗∗∗^	−0.10 (−0.15, −0.06)^∗∗∗^	−0.11 (−0.16, −0.06)^∗∗∗^
P for trend	< 0.001	< 0.001	< 0.001

**TyG-WHtR per IQR**	**Weak grip strength** (**OR** [**95% CI])**		
	**Model 1**	**Model 2**	**Model 3**

Q1	Ref	Ref	Ref
Q2	1.03 (0.79, 1.34)	1.21 (0.91, 1.60)	1.18 (0.87, 1.58)
Q3	0.92 (0.69, 1.20)	1.24 (0.89, 1.73)	1.22 (0.86, 1.75)
Q4	0.87 (0.66, 1.16)	1.50 (0.98, 2.32)	1.52 (0.95, 2.44)
P for trend	0.2620	0.0816	0.104

**Cumulative TyG-WHtR per IQR**	**Absolute grip strength** (***β*-coefficients** [**95% CI])**		
	**Model 1**	**Model 2**	**Model 3**

Q1	Ref	Ref	Ref
Q2	0.51 (−0.16, 1.17)	−0.26 (−0.95, 0.44)	−0.35 (−1.07, 0.38)
Q3	1.27 (0.59, 1.94)^∗∗∗^	−0.31 (−1.12, 0.50)	−0.60 (−1.46, 0.25)
Q4	1.55 (0.85, 2.24)^∗∗∗^	−1.11 (−2.15, −0.06)	−1.51 (−2.62, −0.40)^∗∗^
P for trend	< 0.001	0.0645	< 0.05

**Cumulative TyG-WHtR per IQR**	**Relative grip strength** (***β***-**coefficients** [**95% CI])**		
	**Model 1**	**Model 2**	**Model 3**

Q1	Ref	Ref	Ref
Q2	−0.15 (−0.18, −0.12)^∗∗∗^	−0.12 (−0.14, −0.08)^∗∗∗^	−0.11 (−0.14, −0.08)^∗∗∗^
Q3	−0.21 (−0.24, −0.18)^∗∗∗^	−0.14 (−0.18, −0.10)^∗∗∗^	−0.15 (−0.19, −0.11)^∗∗∗^
Q4	−0.31 (−0.34, −0.28)^∗∗∗^	−0.19 (−0.23, −0.14)^∗∗∗^	−0.21 (−0.25, −0.16)^∗∗∗^
P for trend	< 0.001	< 0.001	< 0.001

**Cumulative TyG-WHtR per IQR**	**Weak grip strength** (**OR** [**95% CI])**		
	**Model 1**	**Model 2**	**Model 3**

Q1	Ref	Ref	Ref
Q2	0.95 (0.73, 1.24)	1.13 (0.85, 1.50)	1.16 (0.86, 1.57)
Q3	0.93 (0.72, 1.22)	1.28 (0.92, 1.80)	1.41 (0.99, 2.02)
Q4	0.87 (0.65, 1.16)	1.49 (0.96, 2.30)	1.63 (1.01, 2.62)^∗^
P for trend	0.3420	0.0661	< 0.05

*Note:p* values are denoted by the following symbols: “^∗^” for < 0.05, “^∗∗^” for < 0.01, and “^∗∗∗^” for < 0.001.

**Table 4:** Regression analysis results of TyG-WHtR for different subgroup clusters and handgrip strength outcomes.

**TyG-WHtR change subgroups**	**Absolute grip strength (*β*-coefficients [95% CI])**		
	**Model 1**	**Model 2**	**Model 3**

Cluster 1	Ref	Ref	Ref
Cluster 2	1.05 (0.50, 1.60)^∗∗∗^	−0.08 (−0.73, 0.56)	−0.22 (−0.89, 0.45)
Cluster 3	1.56 (0.90, 2.21)^∗∗∗^	−0.89 (−1.86, 0.08)	−1.19 (−2.21, −0.16)^∗^

**TyG-WHtR change subgroups**	**Relative grip strength (*β*-coefficients [95% CI])**		
	**Model 1**	**Model 2**	**Model 3**

Cluster 1	Ref	Ref	Ref
Cluster 2	−0.16 (−0.18, −0.14)^∗∗∗^	−0.10 (−0.12, −0.07)^∗∗∗^	−0.10 (−0.13, −0.07)^∗∗∗^
Cluster 3	−0.27 (−0.30, −0.24)^∗∗∗^	−0.14 (−0.19, −0.10)^∗∗∗^	−0.15 (−0.20, −0.11)^∗∗∗^

**TyG-WHtR change subgroups**	**Weak grip strength (OR [95% CI])**		
	**Model 1**	**Model 2**	**Model 3**

Cluster 1	Ref	Ref	Ref
Cluster 2	0.93 (0.75, 1.16)	1.17 (0.90, 1.52)	1.18 (0.89, 1.56)
Cluster 3	0.86 (0.66, 1.13)	1.38 (0.92, 2.08)	1.43 (0.92, 2.22)

*Note:p* values are denoted by the following symbols: “^∗^” for < 0.05, “^∗∗^” for < 0.01, and “^∗∗∗^” for < 0.001.

**Table 5:** Associations between TyG-WHtR, cumulative TyG-WHtR, and handgrip strength stratified by different factors by Model 3.

Subgroups	Triglyceride–glucose index	Absolute grip strength		Relative grip strength		Weak grip strength	
		Beta (95% CI)	*p*	Beta (95% CI)	*p*	OR (95% CI)	*p*
Male	TyG-WHtR	−1.79 (−2.78, −0.81)	**∼0.001**	−0.10 (−0.15, −0.06)	**∼0.001**	1.78 (1.22, 2.62)	**0.003**
	Cumulative TyG-WHtR	−0.47 (−0.73, −0.22)	**∼0.001**	−0.05 (−0.06, −0.04)	**∼0.001**	1.17 (1.05, 1.29)	**0.003**

Female	TyG-WHtR	−0.89 (−1.67, −0.12)	**0.023**	−0.05 (−0.08, −0.02)	**0.004**	1.17 (0.81, 1.69)	0.409
	Cumulative TyG-WHtR	−0.35 (−0.55, −0.15)	**0.001**	−0.03 (−0.04, −0.02)	**∼0.001**	1.11 (1.00, 1.22)	**0.043**

Age < 60	TyG-WHtR	−2.18 (−3.07, −1.29)	**∼0.001**	−0.11 (−0.15, −0.07)	**∼0.001**	1.58 (0.98, 2.53)	0.059
	Cumulative TyG-WHtR	−0.47 (−0.70, −0.23)	**∼0.001**	−0.04 (−0.05, −0.03)	**∼0.001**	1.15 (1.02, 1.30)	**0.025**

Age ≥ 60	TyG-WHtR	−1.47 (−2.36, −0.58)	**0.001**	−0.08 (−0.13, −0.04)	**∼0.001**	1.68 (1.25, 2.27)	**0.001**
	Cumulative TyG-WHtR	−0.54 (−0.78, −0.30)	**∼0.001**	−0.04 (−0.05, −0.03)	**∼0.001**	1.17 (1.08, 1.27)	**∼0.001**

BMI ≤ 24	TyG-WHtR	−1.02 (−1.85, −0.20)	**0.015**	−0.07 (−0.11, −0.03)	**0.001**	1.30 (0.93, 1.81)	0.126
	Cumulative TyG-WHtR	−0.33 (−0.55, −0.11)	**0.003**	−0.04 (−0.05, −0.03)	**∼0.001**	1.11 (1.02, 1.22)	**0.019**

BMI > 24	TyG-WHtR	−1.06 (−2.00, −1.11)	**0.029**	−0.05 (−0.09, −0.02)	**0.004**	1.37 (0.89, 2.10)	0.154
	Cumulative TyG-WHtR	−0.33 (−0.57, −0.09)	**0.007**	−0.03 (−0.04, −0.02)	**∼0.001**	1.11 (1.00, 1.23)	0.058

With hypertension	TyG-WHtR	−1.20 (−2.15, −0.25)	**0.013**	−0.06 (−0.10, −0.02)	**0.002**	1.34 (0.92, 1.96)	0.125
	Cumulative TyG-WHtR	−0.28 (−0.53, −0.03)	**0.024**	−0.03 (−0.04, −0.02)	**∼0.001**	1,09 (0.99, 1.21)	0.076

Without hypertension	TyG-WHtR	−0.80 (−1.61, 0.02)	0.057	−0.80 (−1.61, 0.02)	0.057	1.34 (0.93, 1.92)	0.118
	Cumulative TyG-WHtR	−0.31 (−0.52, −0.10)	**0.004**	−0.04 (−0.05, −0.03)	**∼0.001**	1.12 (1.02, 1.24)	**0.017**

With diabetes	TyG-WHtR	−0.36 (−3.33, 2.61)	0.810	−0.02 (−0.16, 0.12)	0.764	2.44 (0.58, 0.12)	0.241
	Cumulative TyG-WHtR	−0.47 (−1.20, 0.26)	0.202	−0.04 (−0.08, −0.01)	**0.011**	1.08 (0.77, 1.56)	0.646

Without diabetes	TyG-WHtR	−0.99 (−1.63, −0.36)	**0.002**	−0.06 (−0.09, −0.03)	**∼0.001**	1.31 (1.00, 1.71)	**0.046**
	Cumulative TyG-WHtR	−0.27 (−0.43, −0.10)	**0.001**	−0.03 (−0.04, −0.03)	**∼0.001**	1.10 (1.03, 1.18)	**0.005**

With dyslipidemia	TyG-WHtR	−0.08 (−1.74, −0.02)	**0.044**	−0.05 (−0.09, −0.01)	**0.010**	1.33 (0.93, 1.91)	0.122
	Cumulative TyG-WHtR	−0.31 (0.54, −0.08)	**0.008**	−0.03 (−0.04, −0.02)	**∼0.001**	1.11 (1.01, 1.22)	**0.035**

Without dyslipidemia	TyG-WHtR	−1.11 (−2.02, −0.20)	**0.017**	−0.08 (−0.12, −0.04)	**∼0.001**	1.29 (0.88, 1.89)	0.185
	Cumulative TyG-WHtR	−0.27 (−0.50, −0.04)	**0.019**	−0.04 (−0.05, −0.03)	**∼0.001**	1.10 (1.00, 1.22)	0.051

*Note:* “∼0.001” indicates a *p* value less than 0.001.

## Data Availability

The raw data used in this study are accessible via application through the CHARLS website (https://charls.pku.edu.cn/en/). Processed data from the present analysis can be obtained from the corresponding author upon reasonable request.

## Nomenclature

CHARLS: China Health and Retirement Longitudinal Study
TyG: Triglyceride-glucose
BMI: Body mass index
WC: Waist circumference
WHtR: Waist-to-height ratio
IR: Insulin resistance
TC: Total cholesterol
HDL-C: High-density lipoprotein cholesterol
LDL-C: Low-density lipoprotein cholesterol
IQR: Interquartile range
RCS: Restricted Cubic Spline
ROC: Receiver operating characteristic

## Supporting Information

Supporting Table: Main regression results after multiple imputation.
